# Understanding how virtual reality forest experience promote physiological and psychological health for patients undergoing hemodialysis

**DOI:** 10.3389/fpsyt.2022.1007396

**Published:** 2022-12-14

**Authors:** Chung-Heng Hsieh, Dongying Li

**Affiliations:** ^1^Department of Landscape Architecture, College of Art, Fu Jen Catholic University, New Taipei City, Taiwan; ^2^Department of Landscape Architecture and Urban Planning, School of Architecture, Texas A&M University, College Station, TX, United States

**Keywords:** nature, 360-degree nature video, virtual reality, heart rate variability, dialysis

## Abstract

Growing research has found that exposing patients to forest landscapes through technology improves their health. However, studies on the effects of nature therapy sessions on particularly vulnerable groups that require frequent regular treatment such as patients of chronic kidney disease (CKD) is lacking. This study aims to understand the effects of watching panoramic videos of forest walks through virtual reality (VR) headsets on patients. We also examine the effects of the frequency of virtual exposure to forests on the physiological and psychological parameters of patients undergoing dialysis. Twenty-four dialysis patients with a mean age of 65.11 year underwent a virtual nature intervention over the course of 3 weeks. The intervention consists of 3 numbers of 6-min 360-degree nature videos delivered using VR headsets. We measured heart rate variability (HRV) and heart rate (HR) using continuous electrocardiographic (ECG) monitoring and assessed their emotional states using a questionnaire survey and in-depth interviews. The results showed that the initial 6 min of watching of the panoramic videos through VR headsets resulted in significantly reduced HR and sympathetic nerve activity and increased positive emotional responses. However, repeated VR exposure did not further improve the physiological health of the respondents. Considering these results, the use of VR headsets to watch 6-min nature videos indoors can be used to enhance the positive psychological responses among dialysis and bed-ridden patients. As this study is preliminary, future random controlled trials are needed to compare and determine the best dose, content, and delivery methods of such an intervention.

## Introduction

Chronic kidney disease (CKD) is one of the top global public health concerns affecting about 50 million people (1). Studies have revealed that the global all-age mortality rate from CKD increased by 41.5% (35.2–46.5) between 1990 and 2017 (2), making CKD a global challenge. United States Center for Disease Control and Prevention estimates that more than 15% of US adults have CKD (3, 4). The United States Renal Data System (5) revealed that Taiwan has the highest incidence of end-stage renal disease (ESRD) in Asia, and the dialysis rate in Taiwan has become the highest in the world. The number of hemodialysis patients in Taiwan reached 92,524 in 2019. Taiwan’s Health Insurance Administration made NTD51.78 billion in insurance payouts in 2019. Hemodialysis treatment has become a major medical expenditure for the Taiwan Health Insurance Administration. However, besides disease prevalence, discussions related to the psychological wellbeing and effective mental health interventions for this group is scarce (6).

Patients with CKD are among the most vulnerable for psychological distress and depression (6). The pain and mental load that patients receiving dialysis undergo has been well recognized in the literature (7). Patients under hemodialysis usually suffer from various mental problems (8). Anxiety and depression are just two of the problems commonly observed in such patients (9). A longitudinal study following 159 patients for the outcome of depression and anxiety reported that 36.6% had developed depressive symptoms while 31.8% experienced persistent anxiety (10). Anxiety has been found also to lead to a 26% increase in risk of coronary heart disease (11). Batelaan et al. (12) emphasized that anxiety is a key factor in causing cardiovascular disease (CVD), and CVD has also been proven to be the main cause of mortality with kidney disease (13, 14). The prevalence of anxiety in dialysis patients is 43%, which can seriously affect their quality of life (15, 16).

Psychological intervention such as exposure to nature can help relieve chronic patients of anxiety and other negative emotions (17). The theoretical underpinnings for this are the attention restoration theory (ART) and the stress recovery theory (SRT). The ART suggests that mental fatigue can be relieved by exposure to nature (18). Ulrich et al. (19) highlighted that the restorative influence of exposing participants to nature allowed them to experience a positive shift in their emotional states. Such a theoretical framework has been empirically tested in not only the general public but also clinical populations such as cancer patients (20). The restorative effect of exposure to nature on cardiovascular and mental health has been highlighted by related studies (21–23). This beneficial influence could alleviate stress as indicated by increased heart rate variability (HRV) and other physiological measures (24). Contact with natural elements such as green plants indoors could improve the physical functions of people with mental health problems (25). Indoor horticulture has also proven to significantly improve older adults’ quality of life, anxiety, depression, social relations, physical ability, and cognitive ability (26, 27). Higher levels of physical activity were linked to both duration and frequency of green-space visits (28). Spending 120 min a week in nature was considered to have positive effect on health (29), and visiting nature more than once a week was positively associated with general wellbeing (30). Liao et al. (31) also indicated that regular garden visits had positive effects on mood, social interactions, depression, and agitation in people with dementia because of the multisensory, gentle stimuli of the natural environment. In conclusion, psychological intervention such as contact with nature and green plants indoors could help patients experience a positive shift in their emotional state and thus protect their cardiovascular system. Despite the benefits of nature visits (32–35) for hemodialysis patients who receive treatment indoors, a physical visit to outdoor natural area may not be convenient.

The effects of actual nature could be replaced with simulated nature indoors (36). There is growing research interested in virtual reality (VR), which could provide an alternative to actual natural immersion by generating a simulated environment. Most studies highlighted the beneficial effect of technological nature on the promotion of mental health and creativity (37–42). Huang et al. (43) suggested that forest VR experiences were important for the restoration of physiological wellbeing. Mattila et al. (44) found that participants expressed that the VR environment was generally perceived to be as restorative as the physical forest environments and more fascinating and coherent. In summary, VR experience could affect attitude and behavior intention (45–47), improve anxiety and related disorders (48), and be considered a complementary tool for physical training in patients with CVDs (49). As a result, the potential of enhancing physiological and psychological health by virtual nature exposure may become a flexible and easy-to-implement intervention method for vulnerable clinical populations such as patients requiring hemodialysis.

Among the studies that discussed the psycho-physiological effects of nature by using VR devices (41, 50), the target populations were restricted to students (51, 52), the elderly (41), and mental health patients (31). None of these studies focused on a vulnerable population due to a preexisting physical health condition that requires regular and frequent treatment. In addition, most of these studies were conducted under laboratory conditions (41, 43, 51, 52). Therefore, the ecological validity of the results and the effects of nature intervention in naturalistic environments for patients during treatment is unclear. These knowledge gaps stand as obstacles to the development of low-cost and accessible nature-based intervention programs for such patients. With the development of technology in this field, 360-degree videos with VR headsets offer great potential in providing vivid nature experience for hemodialysis patients who are suffering from anxiety in the dialysis room. In the current study, we aimed to identify changes in physiological health outcome from dialysis patients undergoing dialysis before, during, and after the immersive experience of a natural environment thrice over a period of 3 weeks, and we also investigated the psychological response after watching 360-degree nature videos for 3 weeks. As result, we first hypothesized that there will be significant change in the mental health outcome after watching 360-degree nature videos with VR headsets. Second, we hypothesized that the frequency of watching videos with VR headsets might affect the physiological and psychological response of hemodialysis patients.

## Materials and methods

To assess the effect of watching 360-degree nature videos on physiological and psychological parameters of dialysis patients, a clinical trial experiment was conducted in the spring of 2021 in the dialysis rooms of Fu Jen Catholic University Hospital, New Taipei City, Taiwan. We focused on hemodialysis patients, as a high percentage of them are afflicted with psychological distress and anxiety and depressive disorders (53). Considering the limited mobility of patients under dialysis, this study tries to understand the benefits to their psychological wellbeing and physiological response through the use of nature-based VR experiences while remaining indoors during dialysis. There are two stages in this study. In the first stage, we conducted semi-structured interviews to understand the preferred outdoor recreational activities and environments of participants. In the second stage, we conducted the experiment with physiological and psychological measurements to determine how watching these 360-degree nature videos with VR headsets affected the participants’ mental and cardiovascular health. Our measurements included questionnaires using the Pleasure-Arousal-Dominance (PAD) emotional state model, psychological questionnaires, HRV and heart rate (HR), and data derived from continuous electrocardiographic (ECG) monitoring and interviews with the participants.

### Study population

Following the research protocols from related studies (52, 53), we anticipated a sample size of 24 to 40 participants. With regard to the volunteers, the criteria for selection were as follows: the subject should have had at least 6 months of hemodialysis treatment, legally be at least 20 years of age, currently be a hemodialysis patient, have had no chest or abdomen surgery during the previous 3 months, and have had no retinal detachment or other eye surgery during the previous month [see also the criteria developed by (32)]. Prior to the start of the second stage of the trial, subjects were recruited by a trained clinical research nurse, who had to have been screened by a nurse practitioner certified by the Taiwan Society of Nephrology and the Taiwan Nephrology Nurses Association’s peritoneal dialysis training class. Certified nurse practitioners conducted a mini-mental state examination (MMSE) (54) to ensure that the subjects’ MMSE score was 25 or higher and they were safe to participate in the trial. This study followed the relevant human subject research regulation established in Taiwan, and the study was approved by the Institutional Review Board (IRB) of Fu Jen Catholic University (Project No. C108015). Each participant was given a detailed understanding of the experimental procedures and received a participation fee of NTD300. All participants provided written consent forms.

### Study design

Related studies have shown that nature exposure can have psychological benefits on health (17) and that the restorative influence of nature could lead to a shift toward a positive emotional state in patients (18). VR has been applied in the treatment of various anxiety disorders and mood disorders (48). Currently, immersive experiences such as VR are considered a complementary distraction intervention, and studies have been using it as an adjunct to other treatment to alleviate both physical symptoms such as pain, nausea, blood pressure, HR, and respiration rate, as well as psychological symptoms such as anxiety, anguish, fear, and stress (55, 56).

Dialysis patients usually suffer from both physiological and psychological health problems. Dialysis treatments are usually very repetitive, dull, and slow, leading to patients feeling a sense of emptiness. Oftentimes, patients do not know how to fill that time. In addition, they have to endure physical discomfort during the treatment that causes them inconvenience in their daily lives, which in turn affects their mental health (2). According to the theoretical background mentioned previously, we believe that VR immersion can ultimately improve the physiological and psychological health of patients. However, we still required more information with regard to the type of landscape that dialysis patients preferred. For this, we interviewed the patients to understand their environmental preference and then made panoramic videos for the patients based on their statements.

#### Semi-structured interview

**FIGURE 1:**
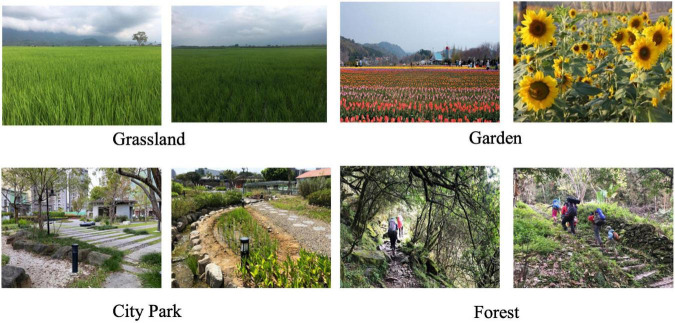
The image provided by research fellows in photo-elicitation interview procedure.

**FIGURE 2:**
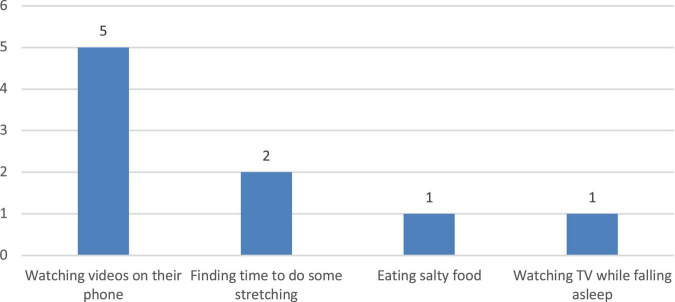
Patients’ common activity during 4 h hemodialysis treatment.

**FIGURE 3:**
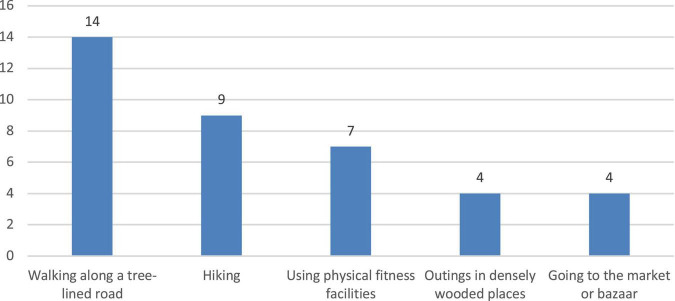
Preferred outdoor recreational activity, as found in interviews with hemodialysis patients.

The first stage of the study focused on understanding the preferred outdoor recreational environments and activities of the participants. The interview results were used as a reference for the researchers when selecting the natural environment. In the first stage, the study used photo-elicitation interviews, which were conducted in previous studies (57–59), to collect participants’ preferences of outdoor recreation activities and settings. Interview content was divided into (1) leisure activities that the patients engaged in during treatment (Figure 1) and (2) outdoor recreational activities and areas preferred by patients (Figure 2). Referring to Kaplan’s definition of environmental preference and ART (60, 61), research fellows selected a wide range of different photographs of outdoor environments to aid the respondents to describe their preferences (Figure 1). The duration of each interview was 20–30 min. The results of interview showed that the participants usually watched videos on their smartphones during their 4-h dialysis treatment (Figure 2). The research fellows also encouraged the participants to describe their experiences of coming into contact with nature. The majority of the participants interviewed mentioned that they had pleasurable experiences with family or friends in more natural environments such as forest trails and preferred to take a walk or hiking in the forest (Figure 3). The results showed that subjects preferred outdoor recreational activities that included walking along a wooded route and densely wooded areas and hiking in the suburbs. Overall, the preferred outdoor recreational activities for dialysis patients were outdoor walks in the suburbs of the city in a forested environment with trees.

#### Site selection and panoramic video production

**FIGURE 4:**
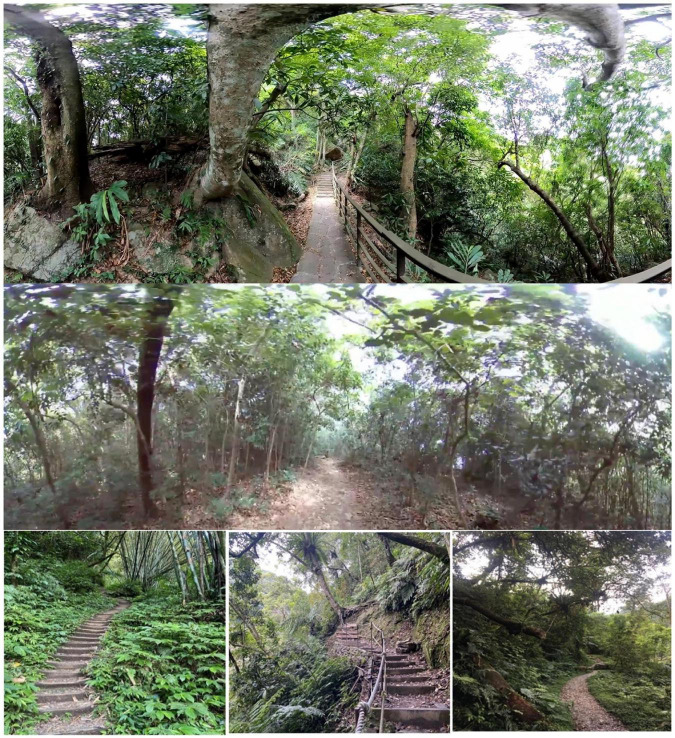
The source image of panoramic video.

Based on the interviews and the purpose of the study, the research team selected three trails within the same area of a forest close to suburban areas as the focus of the panoramic video (Figure 4). The study area was composed of a secondary forest that was covered with a variety of vegetation, such as Oldham’s bamboo (*Bambusa oldhamii)*, Giant Elephant’s Ear [*Alocasia macrorrhiza (L.) Schott*], and Parasol leaf tree (*Macaranga tanarius*). We chose three forest trails with similar landscapes. The three trails featured mild slopes that are easily traversable by the general public. Our research assistants filmed these forest trails using panoramic cameras and a handheld stabilizer. The camera stabilizer could absorb bumps and shakes if the camera was jostled or moved over uneven surfaces to ensure that the shots still appeared smooth. The videos were shot at a height of 165 cm and a walking speed of 3 km/h, mimicking a recreational hiking experience wherein a person may slow down and pause along the trail. However, designated still shots were not included in the videos. The video photographers also put the camera in front of them so that they themselves would not appear in the videos and possibly distract the patients. To avoid any people from appearing in these videos, we asked trail users to wait for a few minutes and kept a distance from these people as much as possible (at least 50 m) before shot these videos so that there were no people in these videos. After the videos were shot, video stitching editing was used to stabilize the image and minimize any shaking. Finally, three panoramic videos featuring similar landscapes (mild forests with slopes) were made. Each of the videos had a duration of about 2 min. The three videos were combined into 1 number of 6-min 360-degree video in this study. To avoid the influence of other attractive landscape elements on the psychological response of the subjects, trails with bodies of water, such as lakes and streams, were excluded from the study. In addition, to minimize the influence of varying ambient and atmospheric conditions such as sunlight, the study was conducted between 11 a.m. and 3 p.m., excluding rainy and cloudy days.

#### Experimental procedure

In preparation for this experiment, the patient volunteers were asked to avoid any unusual medications and alcohol in the 24 h before each experiment session. If they had to take medication, the researcher had to be informed first to facilitate the assessment of suitability for study participation. While all the participants were not habitual smokers, they were all requested to not smoke or consume caffeine in the 2 h prior to the experiment; this was done after we consulted with a nephrologist about the anxiety situation if participants were asked not to smoke or drink coffee. The participants were also asked to avoid using the toilet during the experiment. The status of most of these potential momentary confounders was rechecked at the beginning of the experiment *via* a short interview conducted by research fellows and a confirmation by a clinical research nurse on each participant’s health condition.

**FIGURE 5:**
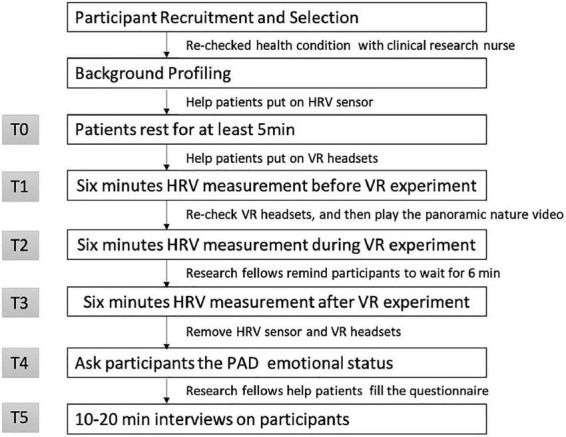
Flow diagram for data collection in 360-degree nature video experiment.

The experiment was conducted every morning starting at 8 a.m. At the beginning of the experiment, the nurse helped research fellows confirm the mental health condition of all participants. As dialysis patients are on hemodialysis for 4 h at a time, they usually start feeling anemic and dizzy by the third to fourth hour. Data collection was mainly done during the first hour of the treatment period, taking into account the physical condition of a patient. First, the patient volunteer was equipped with a portable ECG device and then a VR headset. After the participant had rested for at least 5 min (T0, Figure 5), their HR and HRV were measured (6 min pre-test measurements, T1, Figure 5). Afterward, the patient volunteer was invited to view the 360-degree nature video with VR headsets for 6 min. Their HR and HRV data were measured continuously (6 min mid-test measurements, T2, Figures 5, 10). After the video was viewed, researchers removed the VR headset and continued to measure physiological data for 6 min (post-test measurements, T3, Figure 5). It was important to avoid anemia or emotional problems that could affect the accuracy of the values. Past literature (62–64) showed that an experimental time of 6 to 15 min is less likely to cause discomfort. The study therefore used 6 min as the time for measuring the subjects’ emotions. As the subject had to stay in bed with movement restrictions during hemodialysis, the participants were also informed that they were allowed to rotate their heads for viewing more of the visual field freely. Finally, all equipment was removed. A researcher-assisted questionnaire was administered, and, with the consent of the subjects, research fellows helped participants fill out the questionnaires of the PAD emotional state scale (T4, Figure 5). This protocol was identical for each session. The session consisted of six periods, corresponding to specific measurements or tasks (T0–T5, as illustrated in Figure 5). The experimental procedure above was conducted every week. The participants continuously had their ECG measurements taken, watched the same 360-degree videos, and filled the questionnaire thrice.

**FIGURE 6:**
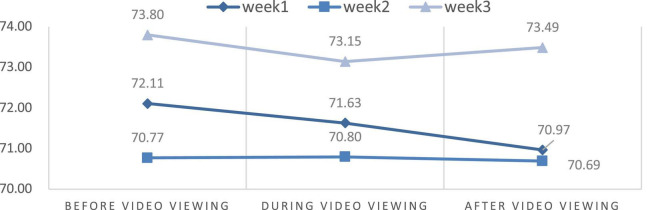
The mean change of heart rate (HR) parameter.

**FIGURE 7:**
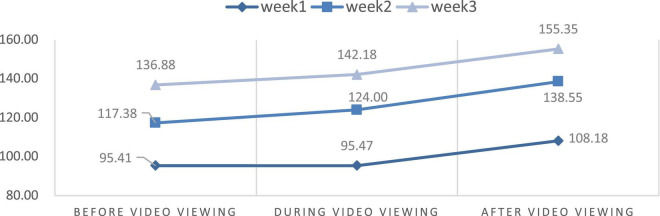
The mean change of high frequency (HF) parameter.

**FIGURE 8:**
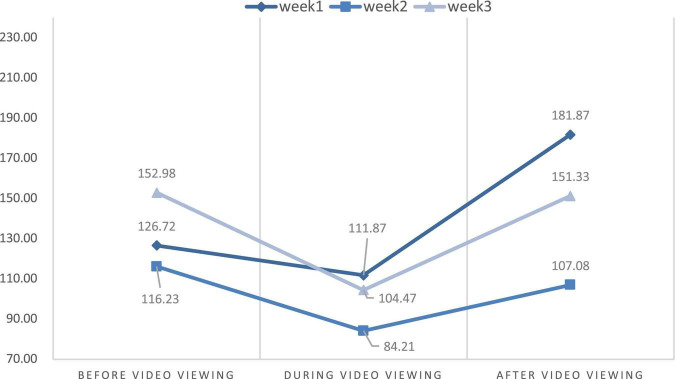
The mean change of low frequency (LF) parameter.

**FIGURE 9:**
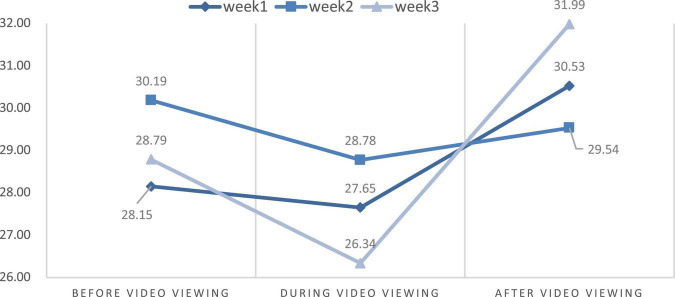
The mean change of SDNN parameter.

**FIGURE 10:**
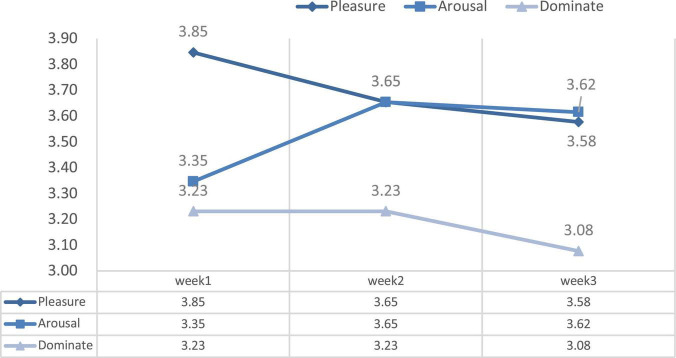
The change of pleasure-arousal-dominance (PAD) emotional status scale in 3 weeks.

#### Measurements

In this study, personal background information including age, work status, and experience with VR equipment was obtained during the experimental phase of the panoramic video. Patient volunteers were asked to answer the MMSE questionnaire, which served as a measure for self-perceived mental health (64). Both the background information and MMSE questionnaire were completed just once before the beginning of the first week. HRV data from continuous ECG measurements and the data from PAD emotional state questionnaires and interview were obtained to determine whether the panoramic video had a positive effect on dialysis patients. The procedure was performed thrice, once per week.

Heart rate variability measures were used to express the interaction between the sympathetic and parasympathetic nervous systems with reference to cardiovascular function. In brief, the sympathetic nervous system is described as dominant in times of anxiety, while the parasympathetic nervous system is dominant in periods of relaxation (65). HRV parameters were calculated in view of the beat-to-beat HR signal and described the variability of the inter-beat intervals (66), which has been used in previous studies on nature exposure and mental health (67, 68). In this study, lightweight portable ECG Holter monitors (HR sensor, BeneGear Inc., New Taipei City, Taiwan), certified by the Taiwan Food and Drug Administration, were used to collect the HR and HRV data. The device was directly attached to one of the 3 electrodes on the participants’ left or middle chests. Considering the device measured 59 mm × 31 mm × 10.7 mm and weighed only 12 g, we assume that the devices had very minimal influence on the participants’ responses. Continuous ECG recordings with a sampling frequency of 125 Hz were derived that is suitable for HRV calculations (69). The devices started recording after being connected to the participants and recorded continuously throughout the entire experiment. Research fellows could view the data recording status by using the BeneGear HRV software with a mobile device in real time, which could help reduce the disturbances of technical or physiological origins.

The overall experimental duration for wearing the sensors was 23 min (T0–T3), the 5 min (T0) of wearing it at the beginning was not included in data analysis. Given that HRV signals can contain disturbances of technical (poorly attached electrodes) or physiological origin (ectopic beats) (70), data processing included manual correction. Such manual corrections were manually checked by means of the software Kubios Premium version 3.5.0 (71, 72) and subsequently by hand in the event that the automatic correction provided by the software to detect the existing artifacts. The frequency domain parameters selected for analysis were high frequency (HF; 0.15–0.40 Hz), with high values showing parasympathetic dominance; low frequency (LF; 0.15–0.40 Hz), where high values indicated sympathetic dominance; and the ratio between LF and HF (LF/HF ratio). From the time domain, we analyzed the standard deviation of the normal inter-beat intervals (SDNN), reflecting the overall HRV (73, 74). The normal range for SDNN is ≥ 30, and the normal value of LF/HF is between 0.5 and 2.0. An index greater than 2 indicates hyperactivity and anxiety; an index less than 0.5 may indicate depression and low mood. HR data were also derived from the ECG signals, with lower values indicating a more relaxed state and higher values indicating a more anxious state during the measurement.

We used the PAD emotional state model to measure the psychological response from the participants. The PAD emotional state model was proposed by Mehrabian (75). The model suggests that emotion has three dimensions. P stands for pleasure-displeasure, which represents the positive and negative characteristics of an individual’s emotional state (i.e., the degree of positivity or negativity). A stand for arousal-no arousal, which indicates the degree of physiological activity of the individual, and alertness is related to the degree of body energy associated with the emotional state. D stands for dominance-submissiveness, which indicates the individual’s control over the situation and others, the individual’s control and influence over others and the external environment, and the degree of subjective control the individual has over their emotional state (76).

With reference to previous studies (75–77) and taking into account the subjects’ unstable physical and psychological conditions during the dialysis procedure, the research fellows asked 10 participants to answer the Chinese Version of the abbreviated PAD emotion scale (78) before and after the VR experience and select the items of the questionnaire that could show the most significant difference with the analysis of paired *T*-test among the means before and after the VR nature experience; this simplified version of three questions related to the PAD emotion scale was then used (Table 1). These questions were asked at the end of the experiment, after removing all the instruments, while the subject had a chance to express their personal opinion and during the PAD questionnaire response period (T4).

**TABLE 1:** Pleasure-arousal-dominance (PAD) emotional status questionnaire items.

Dimension	Questionnaire items	Scale
Pleasure	(1) How pleasure do you feel in this experience	Likert five-point scale
Arousal	(2) How awake do you feel in this experience	
Domination	(3) How easy do you feel in this experience	

Mehrabian (75).

After each experiment, the researchers asked the subjects about their feelings toward the VR experience but only if they wished to express their feelings (T5).

### Data analysis

The data analysis included changes in physiological and psychological data. Physiological changes in the continuous measurement (HR and the HRV parameters) were calculated by comparing the mean values of the pre-experiment period (T1), mid-experiment period (T2), and post-experiment period (T3). The repeated measures ANOVA was used to analyze the statistical significance of physiological changes in the outcome variables before, during, and after the video watching per experiment. Two-way repeated measures ANOVA was used to analyze the statistical significance of the data before and after the video watching among three experiments. Meanwhile, the psychological changes were analyzed by the weekly PAD emotional state model questionnaire. Descriptive statistics were used to understand the mean and standard deviation of emotional states. The repeated measures ANOVA was used to analyze the differences in the PAD emotional states of the subjects each week. Significance for the repeated measures ANOVA was considered at the *p* < 0.05 level. All statistics were performed in IBM SPSS Statistics 24.0 (IBM Corporation, Armonk, NY, USA).

## Results

Initially, 26 participants were enrolled in this 3-week experiment, including 15 females and 11 males. The mean age was 65.11 years. A total of 9 were employed, while the other 17 were unemployed. None of the participants had had any experience with VR headsets and panoramic videos prior to the experiment in this study. All participants completed the experiment during the first and second weeks. The HRV equipment malfunctioned for 2 of the participants in the first week, preventing the collection of physiological values and resulting in the sample data of these 2 participants being deleted, thus leaving 24 samples. The same incident recurred in the second and third weeks for 1 of the participants, finally leaving only 23 samples.

### Changes in physiological stress and psychological state

This study aimed to discuss the effects of VR experience on the level of stress measured using HR and the power of HRV. We also compared the differences in HR and HRV parameters from the first to the third week through the repeated measures ANOVA. Table 2 and Figures 6–9 show the mean values of the assessed physiological parameters, including HR and HRV parameters in the three phases of the experiment during these 3 weeks.

**TABLE 2:** The repeated measure ANOVA of HR and HRV parameters among three phases in 3 weeks.

Parameter	Time	Phase	Mean	*F*-value	*P*-value	*Post-hoc*
HR	Week 1	Before video watching (1)	72.11	3.669	0.033*	1 > 3
		During video watching (2)	71.63			
		After video watching (3)	70.97			
	Week 2	Before video watching (1)	70.77	0.026	0.975	
		During video watching (2)	70.80			
		After video watching (3)	70.69			
	Week 3	Before video watching (1)	73.80	1.678	0.198	1 > 2
		During video watching (2)	73.14			
		After video watching (3)	73.49			
HF	Week 1	Before video watching (1)	95.40	0.965	0.389	
		During video watching (2)	95.47			
		After video watching (3)	108.18			
	Week 2	Before video watching (1)	117.38	0.275	0.761	
		During video watching (2)	123.99			
		After video watching (3)	138.55			
	Week 3	Before video watching (1)	136.88	0.306	0.738	
		During video watching (2)	142.18			
		After video watching (3)	155.35			
LF	Week 1	Before video watching (1)	126.71	2.330	0.109	
		During video watching (2)	111.87			
		After video watching (3)	181.87			
	Week 2	Before video watching (1)	116.23	1.543	0.225	1 > 2
		During video watching (2)	84.21			
		After video watching (3)	107.08			
	Week 3	Before video watching (1)	152.98	1.753	0.185	
		During video watching (2)	104.47			
		After video watching (3)	237.12			
LH/HF	Week 1	Before video watching (1)	1.76	0.309	0.736	
		During video watching (2)	1.63			
		after video watching (3)	1.77			
	Week 2	Before video watching (1)	1.45	0.124	0.884	
		During video watching (2)	1.41			
		After video watching (3)	1.53			
	Week 3	Before video watching (1)	1.37	0.089	0.915	
		During video watching (2)	1.41			
		After video watching (3)	1.46			
SDNN	Week 1	Before video watching (1)	28.15	1.255	0.295	
		During video watching (2)	27.65			
		After video watching (3)	30.53			
	Week 2	Before video watching (1)	30.19	0.148	0.863	
		During video watching (2)	28.78			
		After video watching (3)	29.54			
	Week 3	Before video watching (1)	28.79	2.223	0.119	
		During video watching (2)	26.34			
		After video watching (3)	31.98			

*P < 0.1, **P < 0.05, ***P < 0.01.

As to the changes of physiological parameters, the descriptive statistics showed that the VR nature experience have positive influence on HR and HRV parameters. The subjects’ physiological data showed a slight decrease in HR value before and after watching the video each week, while all HRV values were slightly increased in each week (Figures 6–9), thus showing that VR experiences have positive influence on HF in the three phases of the experiment (Week 1, HF: Mean = 108.18; Week 2, HF: Mean = 138.55; Week 3, HF: Mean = 155.35, Table 2).

We also measured the average changes in the PAD emotional states after experiencing VR for 3 weeks. Table 4 and Figure 10 illustrate the changes in the subjects’ PAD emotional states after the video-watching experience. Descriptive analyses of the results of this questionnaire showed that the mean of the three dimensions was greater than 3 points (Likert five-point scale; 1 = not at all, 5 = very much), indicating that the subjects had a positive response to the immersive experience in all three dimensions. Pleasure scored the highest (*M* = 3.69) and dominance the lowest (*M* = 3.18) among the weekly averages.

**TABLE 3:** The one-way ANOVA of the PAD emotional state scale.

Dimension	Variable	Mean	SD	df	Mean square	*F*-value	*P*-value
Pleasure	Week 1	3.85	0.46	2	1.167	2.006	0.142
	Week 2	3.65	0.69				
	Week 3	3.58	0.76				
Arousal	Week 1	3.35	0.98	2	0.628	0.757	0.473
	Week 2	3.65	0.69				
	Week 3	3.62	0.75				
Dominate	Week 1	3.23	0.43	2	0.821	1.860	0.163
	Week 2	3.23	0.51				
	Week 3	3.08	0.74				

**TABLE 4:** The two-way repeated measures ANOVA of different week and physiological parameters before and after watching video.

Parameter	Variable	df	Mean square	*F*-value	*P*-value
HR	Time (week 1, week 2, week 3)	2	21.421	1.046	0.361
	Phase (before, during, after watching)	1	7.875	2.751	0.114
	Time × Phase	2	7.826	4.361	0.020**
HF	Time (week 1, week 2, week 3)	2	18849.152	0.477	0.624
	Phase (before, during, after watching)	1	13381.210	1.192	0.289
	Time × Phase	2	1913.521	0.198	0.821
LF	Time (week 1, week 2, week 3)	2	33052.207	0.693	0.506
	Phase (before, during, after watching)	1	5154.852	0.432	0.519
	Time × Phase	2	29207.819	2.347	0.109
LF/HF	Time (week 1, week 2, week 3)	2	1.524	0.590	0.559
	Phase (before, during, after watching)	1	0.161	0.174	0.681
	Time × Phase	2	0.249	0.427	0.656
SDNN	Time (week 1, week 2, week 3)	2	4.363	0.012	0.988
	Phase (before, during, after watching)	1	110.573	1.072	0.313
	Time × Phase	2	22.676	0.267	0.767

*P < 0.1, **P < 0.05, ***P < 0.01.

### Differences of physiological and psychological state among phases and weeks

To compare the differences among the three phases each week and understand the effect of the experience on physiological response, repeated measures ANOVA was used. The statistical results showed that the immersive experience of VR has a significant effect on HR among the phases before and after video-watching in Week 1, and there is also a significant effect on HR among phases before and during video-watching in Week 3. In Week 1, the HR value were significantly different among the phases before and after video-watching [*F*_(2,22)_ = 3.669; *P* = 0.033]. The HR value after video-watching (Mean = 70.97) was significantly lower than the value before video watching (Mean = 72.11). In Week 3, the HR values were significantly different among the phases before and after video-watching [*F*_(2,22)_ = 1.678; *P* = 0.198]. The HR value during video-watching (Mean = 73.14) was significantly lower than that before video watching (Mean = 73.80).

With respect to the differences in HRV parameters among the 3 phases in the 3 weeks, LF values among 3 phases were significantly different in Week 2 while other HRV parameters among 3 phases were significantly different in three weeks. The LF value during video-watching (*M* = 84.21) was significantly lower than that before video-watching (*M* = 116.23) (Table 2). In the last week, there was no significant difference before, during, and after video-watching in the values of all HRV parameters—the HR value slightly decreased while the other values of physiological parameters increased slightly.

Additionally, we presume that there was significant difference of HR and HRV parameters between the mean of the phase before and after the video-watching. We thus used the two-way repeated measures ANOVA to test the significant difference of the HR and HRV mean before and after video watching among 3 weeks. The statistical results showed that the interaction of phase and time have significant effort on the difference of HR value between the phase before and after video watching among 3 weeks (*F* = 4.361; *p* = 0.020 < 0.050; Table 3) while there was no significant effort on all HRV parameters (Table 3). The difference and range of the HR value in the phases before and after video-watching in Week 1 (Mean before video watching = 72.11; Mean after video watching = 70.97) was more significant than in Week 2 (Mean before video-watching = 70.77; after video-watching = 70.69) and in Week 3 (Mean before video-watching = 73.80; Mean after video-watching = 73.49), indicating that viewing the same 360-degree video thrice would not increase the physiological benefits (Table 2).

In terms of psychological state, the results of the repeated measures ANOVA over 3 weeks showed that there were no significant differences within the three emotional state parameters (pleasure dimension: *p* = 0.142 > 0.05; arousal dimension: *p* = 0.473 > 0.05; Dominate dimension, *p* = 0.163 > 0.05). Above all, the arousal dimension value in Week 1 was the lowest (Mean = 3.35), while in Week 2 it was the highest (Mean = 3.65). The pleasure and dominance mean scores were the highest in Week 1 (Mean = 3.85; Mean = 3.23) and then gradually decreased with increasing frequency over the 3-week period (Table 4).

### The findings from interviews with participants

After completing the bio-response and questionnaire survey, participants were interviewed to better understand their perceptions of watching 360-degree nature video with VR device In Week 1, 21 participants were willing to answer the interview question, while 3 were not. In Weeks 2 and 3, there were 16 participants who agreed to be interviewed while 7 did not due to a low state of health after the dialysis treatment. Those who were willing to be interviewed responded positively to the VR experience. After the first week of the experiment, the 360-degree nature video provided content with a sense of presence, which enhanced the participants’ positive emotions and reactions. This indicated that the VR experience had increased their willingness to encounter nature.

*“I enjoyed that feeling of walking from start to finish and the high level of interaction.” —P2 (Week 1)*

*“The video feels similar to the walking I used to do.” —P4 (Week 1)*

*“I like that it feels like I am walking really. The trees and water in the video are very nice, as well the call of the insects and birds.” —P5 (Week 1)*

*“It was good that some videos were interspersed with specific spots or walking content.” —P6 (Week 1)*

*“I don’t go out much anymore, so watching these videos has triggered my desire to go out and take a walk.” —P14 (Week 2)*

*“The images are very realistic and are similar to my own experience of going out for a walk. Sometimes they are exactly the same.” —P16 (Week 2)*

Most of the patients who were willing to be interviewed responded positively to the experience. Only a few felt dizzy after wearing the VR headset or began to experience other uncomfortable physical sensations after wearing it a few times. In addition, some respondents thought that just experiencing a walk in the forest was a bit boring. These factors resulted in a small number of respondents feeling bored after repeated exposure to the scene, which has been discussed in the section on limitations of this study.

*“The picture is a little too close to the screen. It makes me feel a little discomfort.” — P1 (Week 3)*

*“The walking pace in the video is a bit slow, which is different from my walking speed. If I watch too long, I’ll feel dizzy.” —P5 (Week 3)*

*“I feel like I’m walking every week, so I don’t get to interact with other people. It makes me feel a bit lonely and bored.” — P18 (Week 3)*

*“I feel like there is no interaction after watching for a long time. The equipment is heavy, and I get a little dizzy.” —P20 (Week 3)*

## Discussion

### Findings

The current study aims to understand the effect of watching 360-degree nature videos with VR devices and confirm the influence of duration and frequency of VR nature exposure on health outcomes. Lanki et al. (24) found that at least 15 min of sedentary watching could result in beneficial short-term changes in cardiovascular risk factors. Kabisch et al. (32) suggested that a relatively narrow period (of 2 weeks) of exposure could help minimize the influence caused by the participants’ social context conditions. We presented the results from a clinical trial experiment in which we obtained physiological and psychological health data from 24 dialysis patients before, during, and after a VR experience over a period of 3 weeks. Participants were asked to watch the VR video by using a device for 6 min thrice. We thus investigated the effects of short-term exposure on physiological and psychological parameters relevant for cardiovascular health.

The results showed a significant decrease in HR between the phases before and after the experience in Week 1. It means that short-term watching of 360-degree video with VR headsets could help reduce stress during a dialysis procedure. However, changes in HRV parameters were not significant in Week 1. Briki and Majed (79) revealed that walking for 20 min in a green environment induced a significant reduction in HR values. Li et al. (80) also found that nature videos could buffer HR acceleration. In the current study, participants had to lie in bed for their treatment, which restricted their movement. Thus, we could only design the 6-min 360-degree nature video with VR headsets for the patients. The results showed that for the 6-min duration, the video could significantly improve mental health and then decrease HR. This corroborates the calming and relaxing effect of green color on human beings. The LF value that describes sympathetic nerve activity decreased significantly between the phase before and during experience in Week 2, while other parameters did not have a marked change. The decrease in LF demonstrates that our video with VR headsets helps participants reduce their negative emotions, which is beneficial for the autonomic nervous system. Saadi et al. (81) found that after visiting an outdoor environment for 45 min, a decrease in the mean LF/HF ratio was observed. Tsunetsugu et al. (82) indicated that watching urban forest landscapes for 15 min could also significantly lower the LF of young and healthy males. Our study showed that watching 360-degree nature videos with VR devices might have relaxing effects. Furthermore, we speculated that the cardiovascular health of patients who are receiving dialysis treatment improved due to the improvement of their mental health.

Our study highlighted the short-term health benefits that patients who were undergoing dialysis treatment received from watching panoramic nature videos using a VR device. However, our study still showed that repeated weekly watching of videos in a dialysis room did not have a positive influence on health and wellbeing. The change in the psychological responses in our study also revealed that the second and third watching did not have an effect on mental health. In the first week of the experiment, the participants registered the highest pleasure and dominance scores toward the panoramic nature video; however, after the experiment in the third week, pleasure and dominance emotion declined to their lowest points. Only the arousal feeling increased progressively with the experiments over three weeks. Furthermore, the interviews with participants also showed that they experienced the most positive responses from the experiment in first week. Most of the negative responses were brought up from the experiment in Week 3. Above all, our study provided admissible evidence that short-term watching of 360-degree nature videos has a benefit on mental health as found in similar studies (83, 84), while more diversity in landscape may help reduce boredom and provide long-term benefits.

### Strengths and limitations

The current study adds to previous research in three ways. First, we included patients undergoing dialysis of both sexes with a mean age of 65.11 years. Other studies have focused on healthier, happier subjects such as students (84), young males (83), young females (81), and elderly adults (41). Second, we introduced 6-min VR nature videos indoors as a medium that could provide physiological and psychological benefits that might be valuable for health care. Third, while making the 360-degree video for this study, efforts were made to provide an immersive nature experience as realistically and authentically as possible. We controlled the height of the shot at eye level and the walking speed at 3 km/h. We also provided participants with the high-definition VR headsets to view the videos. Also, our study protocol was highly standardized concerning the experimental procedure, and the same protocol was maintained each week.

While this study deepens our understanding of the potential value of VR nature to health and wellbeing, the results are preliminary and need to be interpreted with caution. First of all, this study employs a within-subject design and does not involve a control group. This is due to the challenges involved in recruiting patients receiving hemodialysis treatment who are willing to participate in this study during the same timeframe. Future studies can include a control group with no video watching, or viewing a barren or other types of VR scene, to strengthen the results.

Second, we encountered participant drop-out issues during the experiment. Previous studies (53, 54) indicated that the patients usually had negative emotional responses in the course of dialysis treatment. Considering that the participants were dialysis patients, several participants also felt emotionally unstable during the dialysis process and were advised by our clinical research nurse to discontinue their participation in the experiment. This led to the decrease in the effective sample size. We adopted the technique previously used regarding the inclusion of an interview (41) to acquire additional understandings of the reasons for drop out and experience of all participants. Fortunately, the result of questionnaire surveys and interviews provided credible evidence to the psychological health of watching 360-degree nature videos.

Third, this study aims to establish preliminary evidence regarding physiological and psychological response from patients receiving repeated 360-degree video intervention over the course of 3 weeks. As such, it was vital that participants watched the same video three separate times, so that we could accurately compare the physiological and psychological responses each time. However, as the results suggested, repeated exposure to the same video might result in the patients feeling accustomed to it or less engaged, leading to reduced effects. As this is a limitation that requires attention for all interventions with immersive technology, programmed variations that better mimic the unexpected encounters in actual green space may be helpful. Therefore, building on our results, future studies may need to consider qualitative differences in the VR nature experience to alleviate the fatigue issue.

Additionally, although measures were taken to stabilize the video during shooting and processing, a few participants still reported discomfort wearing the headset or dizziness during the viewing. Such discomfort has been recognized in VR-based studies, which may be due to sensory conflicts. The nature intervention selected in this study is a mild hiking experience based on preference survey, which may be related to the reported dizziness issue and therefore involved forward movement and vertical movement at times.

Finally, hemodialysis patients have to receive treatment twice a week under normal conditions. The experiment could be performed within 2 weeks ideally. Due to limitations in terms of staff, time, and restrictions due to the pandemic, the number participants who were available for a fixed period of time within 2 weeks was very few. We had to extend the experiment period to 3 weeks, which made it hard to control for the impact of social context conditions on health response. According to other study protocols (32, 85), we acknowledge the resulting limitations by identifying potential significant within-person changes in health outcomes every week and also looking at the difference of mean values across different weeks. Indeed, we could not recruit a full representative sample that might potentially limit the generalizability of this study. We also had to trust in the information provided by the participants regarding medication intake, and we cannot guarantee its accuracy. In addition, the lifestyle habits of participants could not be monitored by the research fellows throughout these experiments in 3 weeks, which could have had some impact on the physiological responses.

## Conclusion and outlook

This study investigated the effects of short-term exposure to nature videos through a VR experience on physiological and psychological health parameters of patients undergoing dialysis. We found decreases in HR and LF parameters in the first 2 weeks, indicating potentially positive effects on cardiovascular health. However, we found slight increases in HF parameters after watching the same 360-degree nature video in 3 weeks. indicating adverse effects on cardiovascular health as well as detrimental effects on the effectiveness of preventive measures.

We proved that watching the 360-degree nature video with VR headsets for 6 min could provide short-term physiological and psychological health benefits for hemodialysis patients. Related studies (28, 29) have highlighted the benefits of duration and frequency when it comes to nature exposure for patients. However, we found that repeated viewings of the same panoramic videos were not likely to keep increasing the health benefits. As a result, we suggest that future studies might discuss the effects of repeated sessions with other similar landscapes or different landscapes (such as forests, rivers, oceans, mountains, etc.) on mental health. In addition, we found that excessive watching of nature videos using VR might lead to negative responses. Further investigations would be required to apply the VR techniques for long-term health outcomes for hemodialysis patients. Based on these findings, watching various 360-degree nature videos could be considered a valuable invention for promoting cardiovascular health during dialysis procedures. The opportunity to experience nature with a VR device could increase a patient’s intention to commune with nature in the future.

On an individual level, adopting a lifestyle of spending time in nature is generally related to the hobby development of outdoor recreational activities of dialysis patients who are usually affected by the accessibility and size of green spaces near urban areas. The reserve of large-scale public green areas such as urban forests or forest parks is essential for the development of urban and health care. Thus, medical therapy units could have the chance to provide a technical nature experience for patients with dialysis to increase their intentions toward nature exposure. Future work could concentrate on the creation of technological nature and the identification of specific natural elements such as water, trees, grasslands, and mountains that would attract dialysis patients, allowing them to be physically active and reduce anxiety while undergoing a dialysis procedure (86). All of this will be helpful from the perspective of creating supportive landscape interventions for the better health care of dialysis patients.

## Data availability statement

The original contributions presented in this study are included in the article/Supplementary material, further inquiries can be directed to the corresponding author.

## Ethics statement

The studies involving human participants were reviewed and approved by the Institutional Review Board (IRB) of Fu Jen Catholic University (Project No. C108015). The patients/participants provided their written informed consent to participate in this study.

## Author contributions

C-HH: conceptualization, methodology, formal analysis, investigation, validation, data curation, writing original draft, project administration, and funding acquisition. DL: review and editing original draft. Both authors contributed to the article and approved the submitted version.
